# Osteomyelitis of Parietal Bone in Melioidosis

**DOI:** 10.3201/eid1308.070479

**Published:** 2007-08

**Authors:** Nina G. Miksić, Nadja Alikadić, Tatjana Z. Lejko, Alenka Andlovic, Jernej Knific, Janez Tomažič

**Affiliations:** * General Hospital Maribor, Maribor, Slovenia; † University Medical Center Ljubljana, Ljubljana, Slovenia; ‡ University Ljubljana, Ljubljana, Slovenia

**Keywords:** melioidosis, osteomyelitis, fever, Slovenia, letter

**To the Editor:** In Europe and the United States, melioidosis is a rare disease, with no cases reported thus far from Slovenia. However, it is a relatively common disease in certain areas of Southeast Asia and northern Australia. Potentially fatal, this disease is caused by the gram-negative bacillus *Burkholderia pseudomallei*, an environmental organism found in the soil and water of disease-endemic areas. Human infections are mostly acquired through percutaneous inoculation during contact with contaminated water and soil, although inhalation is also a recognized route of acquisition (*1*). Heavy monsoon rain is associated with severe disease course (*2*). Melioidosis was reported in some persons injured in the Tsunami in 2004 (*3*). The disease has a wide spectrum of signs and symptoms (*4*). Osteomyelitis is a rare manifestation. It occurs in <5% of cases and is a clinical challenge to diagnose and treat (*1*,*4*,*5*).

We describe a case of melioidosis in a previously healthy, 40-year-old Slovenian man. The patient had been working as a basketball trainer in Jordan for the previous 12 months and was traveling to Brunei in mid-summer 2006, 14 days before the illness started. While visiting Brunei, he sustained a minor head trauma when he hit his head on a night table at the hotel. Ten days later, high-grade fever up to 40°C developed, without any other signs or symptoms of disease. After returning to Jordan, the patient was admitted to a local hospital and received different antimicrobial agents without any improvement of his medical condition. After 6 weeks of unsuccessful treatment, he decided to continue medical treatment in Slovenia.

On admission to our hospital, he reported headache and persistent high fever of 6 weeks’ duration. Physical examination indicated high fever (39.5°C) and occipital swelling without any neurologic deficits or other abnormal findings. Initial complete blood cell count, liver function test results, blood urea nitrogen levels, and creatinin levels were normal. C-reactive protein was 60 mg/L, and erythrocyte sedimentation rate was 47 mm/h. Results of chest radiograph and abdominal ultrasound were normal. Results of repeated blood cultures and urinalysis were negative.

The suspected clinical diagnosis was brucellosis (the patient had eaten unpasteurized soft cheese during his stay in Jordan, and brucellosis is endemic in the Middle East). While waiting for *Brucella* spp. tests, we began empirical antimicrobial drug treatment with doxycycline. The patient’s condition improved promptly. He became afebrile after 4 days of therapy. In the following week, ultrasound of occipital area soft tissue was performed, and posttraumatic seroma was diagnosed. *B. pseudomallei* was isolated from the seroma on sheep blood agar and identified with VITEK 2 gram-negative identification card (bioMérieux, Marcy l’Etoile, France). The isolate was sensitive to piperacillin, piperacillin-tazobactam, ceftazidime, imipenem, meropenem, and chloramphenicol. It was resistant to aminoglicosides (gentamicin, tobramycin, amikacin, netilmicin), colistin, and polymyxin B. Etest MIC showed susceptibility to doxycycline (MIC 2 μg/mL) and trimethoprim/sulfamethoxazole (TMP/SMX) (MIC 1/19 μg/mL). Susceptibility of *B. pseudomallei* to TMP/SMX was tested with Etest because the disc-diffusion method is inappropriate and can overestimate the extent of resistance (*6*).

The patient later recalled going on a jungle trip in Brunei the day after his accident. During the trip, he scratched his head, and the skin started to bleed. Thus, he likely inoculated bacteria into the subcutaneous tissue of the head. Fever developed 10 days later.

Computed tomography of the scalp was performed (Figure), and osteomyelitis of the right parietal bone was detected. Magnetic resonance imaging (MRI) excluded involvement of intracranial tissues. Doxycycline was stopped and, as recommended, treatment with ceftazidime and oral TMP/SMX was started. The patient received 8 weeks of intensive parenteral therapy. Once he was discharged, he received 4 months of oral eradication therapy with TMP/SMX and doxycycline. The outcome was excellent. He is now without signs and symptoms of disease, has normal laboratory test results, and has no signs of inflammation on MRI.

**Figure:**
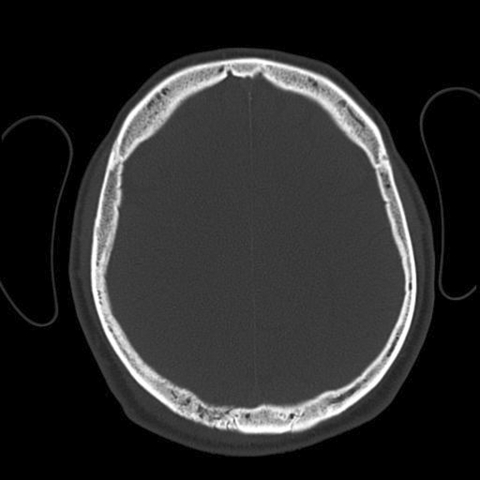
Axial nonenhanced computed tomography showing moth-eaten appearance of right parietal bone characteristic of osteomyelitis.

Involvement of the skin and soft tissue is common in melioidosis (*7*). Osteomyelitis is a rare manifestation, usually part of a disseminated infection involving metaphyseal regions of long bones and vertebral bodies. Localized bone involvement is very rare (*8*). In a Thailand group of 21 patients with musculoskeletal melioidosis, all were initially treated with surgical debridement, followed by long course of antimicrobial therapy (*9*). A single report of parietal bone osteomyelitis was found in the literature; it was connected to a cerebral abscess due to hematogenous dissemination (*10*). Because of the specific location of the osteomyelitis (close to the leptomeninges), nonextensive bone damage, and good initial response to antimicrobial therapy, we decided on conservative therapy only.

Melioidosis, although a rare disease, should be considered in the differential diagnosis of any febrile illness in patients returning from disease-endemic regions, especially Thailand and northern Australia. Without special awareness of this possibility, microbiologic laboratories in nonendemic regions could likely misidentify the bacteria and consequently misdiagnose the organism.
